# Supplementary material for: Evolutionary–developmental (evo-devo) dynamics of hominin brain size
Source: Nat Hum Behav. 2024 May 27;8(7):1321–33. doi: 10.1038/s41562-024-01887-8 (PMC11272587; doi:10.1038/s41562-024-01887-8)
Supplement: Supplementary file 2 — Reporting Summary [file 41562_2024_1887_MOESM2_ESM.pdf]

nature portfolio

Corresponding author(s): Mauricio González-Forero

Last updated by author(s): Apr 10, 2024

Reporting Summary

Nature Portfolio wishes to improve the reproducibility of the work that we publish. This form provides structure for consistency and transparency in reporting. For further information on Nature Portfolio policies, see our Editorial Policies and the Editorial Policy Checklist.

Statistics

For all statistical analyses, confirm that the following items are present in the figure legend, table legend, main text, or Methods section.

n/a	Confirmed	
[ ]	[x]	The exact sample size (*n*) for each experimental group/condition, given as a discrete number and unit of measurement
[x]	[ ]	A statement on whether measurements were taken from distinct samples or whether the same sample was measured repeatedly
[x]	[ ]	The statistical test(s) used AND whether they are one- or two-sided<br>*Only common tests should be described solely by name; describe more complex techniques in the Methods section.*
[x]	[ ]	A description of all covariates tested
[x]	[ ]	A description of any assumptions or corrections, such as tests of normality and adjustment for multiple comparisons
[ ]	[x]	A full description of the statistical parameters including central tendency (e.g. means) or other basic estimates (e.g. regression coefficient) AND variation (e.g. standard deviation) or associated estimates of uncertainty (e.g. confidence intervals)
[x]	[ ]	For null hypothesis testing, the test statistic (e.g. *F*, *t*, *r*) with confidence intervals, effect sizes, degrees of freedom and *P* value noted<br>*Give P values as exact values whenever suitable.*
[x]	[ ]	For Bayesian analysis, information on the choice of priors and Markov chain Monte Carlo settings
[x]	[ ]	For hierarchical and complex designs, identification of the appropriate level for tests and full reporting of outcomes
[x]	[ ]	Estimates of effect sizes (e.g. Cohen's *d*, Pearson's *r*), indicating how they were calculated

Our web collection on statistics for biologists contains articles on many of the points above.

Software and code

Policy information about availability of computer code

Data collection	No data was collected in this study and so no software was used for data collection.
Data analysis	Custom computer code to run the model and generate the figures was prepared to run in Julia 1.7.2. All code is available in the Supplementary Information and in https://doi.org/10.5281/zenodo.10887414.

For manuscripts utilizing custom algorithms or software that are central to the research but not yet described in published literature, software must be made available to editors and reviewers. We strongly encourage code deposition in a community repository (e.g. GitHub). See the Nature Portfolio guidelines for submitting code & software for further information.

Data

Policy information about availability of data

All manuscripts must include a data availability statement. This statement should provide the following information, where applicable:

- Accession codes, unique identifiers, or web links for publicly available datasets
- A description of any restrictions on data availability
- For clinical datasets or third party data, please ensure that the statement adheres to our policy

No data was collected in this study. All data used were previously published in references provided in the main text or supplementary information.

Human research participants

Policy information about studies involving human research participants and Sex and Gender in Research.

Reporting on sex and gender	This study did not involve real human participants. The model considers only (simulated) females.
Population characteristics	This study did not involve real human participants.
Recruitment	This study did not involve real human participants.
Ethics oversight	This study did not involve real human participants.

Note that full information on the approval of the study protocol must also be provided in the manuscript.

Field-specific reporting

Please select the one below that is the best fit for your research. If you are not sure, read the appropriate sections before making your selection.

☐ Life sciences ☐ Behavioural & social sciences ☒ Ecological, evolutionary & environmental sciences

For a reference copy of the document with all sections, see nature.com/documents/nr-reporting-summary-flat.pdf

Ecological, evolutionary & environmental sciences study design

All studies must disclose on these points even when the disclosure is negative.

Study description	This study mathematically models the evolutionary and developmental dynamics of hominin brain and body size.
Research sample	This study did not collect data. It used previously published data of various kinds. First, brain and body sizes for human females over ontogeny were obtained from the spline fit of Kusawa et al (2014) to the cross-sectional data of Dekaban and Sadowsky (1978) for 1963 females from several hospitals in Washington, DC and Bethesda, MD. Second, fossil and extant data for adult brain and body sizes for 11 hominin species were taken from Smaers et al (2021) who took them from Grabowski et al (2015) and Kappelman (1996) among others. Fossil data for adult brain and body size for H. floresiensis were taken from Brown et al (2004) and for H. naledi from Garvin et al (2017). Fossil and extant data for adult brain and body sizes for great apes, lesser apes, cercopithecines, lemurs, and lorises were taken from Smaers et al (2021) who took them from multiple sources. Third, the mathematical model depends on various parameters that were estimated by González-Forero et al (2017) and González-Forero and Gardner (2018) from previously published metabolic, demographic, and neuroscience data with references in such publications.
Sampling strategy	This study did not collect data.
Data collection	This study did not collect data.
Timing and spatial scale	This study did not collect data.
Data exclusions	Failed organisms in Fig. 2a (gray dots) were not included in the calculation of the regression coefficient reported in that figure. The reason is that failed organisms here have a body composed entirely of brain and would thus be inviable.
Reproducibility	All attempts to repeat the model solutions yielded the results reported.
Randomization	This study did not collect data.
Blinding	The data used were blinded by the original publications where the data were taken from.

Did the study involve field work? ☐ Yes ☒ No

Reporting for specific materials, systems and methods

We require information from authors about some types of materials, experimental systems and methods used in many studies. Here, indicate whether each material, system or method listed is relevant to your study. If you are not sure if a list item applies to your research, read the appropriate section before selecting a response.

Materials & experimental systems

n/a	Involved in the study
☒	☐ Antibodies
☒	☐ Eukaryotic cell lines
☒	☐ Palaeontology and archaeology
☒	☐ Animals and other organisms
☒	☐ Clinical data
☒	☐ Dual use research of concern

Methods

n/a	Involved in the study
☒	☐ ChIP-seq
☒	☐ Flow cytometry
☒	☐ MRI-based neuroimaging
